# Towards the Well-Tempered Chloroplast DNA Sequences

**DOI:** 10.3390/plants10071360

**Published:** 2021-07-02

**Authors:** Ante Turudić, Zlatko Liber, Martina Grdiša, Jernej Jakše, Filip Varga, Zlatko Šatović

**Affiliations:** 1 Centre of Excellence for Biodiversity and Molecular Plant Breeding (CoE CroP-BioDiv), Svetošimunska cesta 25, 10000 Zagreb, Croatia; mgrdisa@agr.hr (M.G.); fvarga@agr.hr (F.V.); zsatovic@agr.hr (Z.Š.); 2 Faculty of Agriculture, University of Zagreb, Svetošimunska cesta 25, 10000 Zagreb, Croatia; zlatko.liber@biol.pmf.hr; 3 Faculty of Science, University of Zagreb, Marulićev trg 9a, 10000 Zagreb, Croatia; 4 Biotechnical Faculty, University of Ljubljana, Jamnikarjeva 101, 1000 Ljubljana, Slovenia; Jernej.Jakse@bf.uni-lj.si

**Keywords:** chloroplast genome, genome assembly, standardization, cyclic shift, inversion

## Abstract

With the development of next-generation sequencing technology and bioinformatics tools, the process of assembling DNA sequences has become cheaper and easier, especially in the case of much shorter organelle genomes. The number of available DNA sequences of complete chloroplast genomes in public genetic databases is constantly increasing and the data are widely used in plant phylogenetic and biotechnological research. In this work, we investigated possible inconsistencies in the stored form of publicly available chloroplast genome sequence data. The impact of these inconsistencies on the results of the phylogenetic analysis was investigated and the bioinformatic solution to identify and correct inconsistencies was implemented. The whole procedure was demonstrated using five plant families (Apiaceae, Asteraceae, Campanulaceae, Lamiaceae and Rosaceae) as examples.

## 1. Introduction

Chloroplasts are one of the main specifics of the plant cell. They are organelles specialized for carrying out photosynthesis. It is generally accepted that the existence of chloroplasts in a plant cell is the result of the evolution of a cyanobacterial-like prokaryote that managed to incorporate into a eukaryotic cell as an endosymbiont. Due to their ability to harness solar energy, chloroplasts are responsible for transforming ancient plant cells from heterotrophy to autotrophy [1].

Chloroplasts possess a semi-autonomous genetic system called the plastome [2]. The coding ability of the plastome has been greatly reduced compared to its progenitor, leaving very little of the ancestral gene complement in chloroplasts [3]. In fact, only genes involved in photosynthetic reactions have been retained in the chloroplast, while most other genes have migrated to the nuclear genome, where the majority of chloroplast proteins are encoded and imported posttranslationally into the chloroplast [1]. Accordingly, the expression of plastome sequences is controlled by imported nuclear factors [4].

The chloroplast genome (cpDNA) is a double-stranded DNA molecule of about 120 kb to 160 kb in size that encodes about 100 proteins [5,6]. This genome is very AT-rich, especially in non-coding regions where the AT content can be as high as 80%. Chloroplast genes account for half of the total nucleotide sequence, with the remainder comprising introns, regulatory regions, and intergenic spacers [7]. Early research suggested that the chloroplast genome is circular [8], but recent cytological studies have demonstrated that cpDNA molecules can also be branched and linear [9,10].

The typical structure of the chloroplast genome consists of a large single-copy (LSC) and a small single-copy (SSC) region separated by two identical inverted repeat (IR) regions. Each land plant individual cell contains with equal frequency at least two structural haplotypes (heteroplasmy) that differ in the orientation of the single-copy regions, due to the homologous recombination between IRs [11,12,13]. The genes in the SSC and LSC regions have a higher rate of synonymous substitutions compared to those in IR [14]. The length of the inverted repeats is usually between 10 and 30 kb, but can be up to 76 kb in extreme cases [15]. Probably the most important role of IRs is in maintaining plastome stability [16]. Intramolecular recombination between IR copies has been proposed as a mechanism to prevent divergence between two IR copies and the one responsible for the polarity of the segment between the two copies, a phenomenon known as flip-flop recombination [11,13]. IR loss has been documented in several angiosperm families [2,17].

Although plastids are maternally inherited in more than 80% of angiosperms [18,19,20,21], there is evidence for biparental inheritance (e.g., [18,20]), a mechanism that appears to have evolved to rescue species with plastome-genome incompatibility [14,21,22,23,24,25]. Chloroplast genes are usually completely collinear in most angiosperm taxa. However, there are several cases where inversions, insertions, deletions, and changes in the size of IR regions has resulted in gene sequence reconfiguration [4].

Chloroplast genome sequences have been widely used in plant phylogenetics, phylogeography, and molecular evolution research. They have numerous advantages (simple structure, mostly uniparental mode of inheritance, haploidy, the slow evolutionary rate, etc.) compared to nuclear genomes [26].

Advances in DNA sequencing have increased the affordability of cpDNA sequence data, while the multiplicity of cpDNA molecules [27,28] has increased its ease and usability [29]. It is often possible to assemble a much shorter whole chloroplast genome, while assembly of a whole plant DNA genome of the same species is unattainable. This is reflected in the large number of available cpDNA sequences in public databases [30]. At the beginning of 2021, nearly 5000 plant cpDNA were available in the National Center for Biotechnology Information (NCBI) public genome database.

Several types of bioinformatic approaches have been used in research to assemble chloroplast genome sequences [31], such as (1) in-house implemented pipelines [32,33], (2) standard de novo assemblers, e.g., SOAPdenovo [34] and AbySS [35], and (3) specialized organelle or plastome assemblers, e.g., Fast-Plast [36] and GetOrganelle [37]. Bioinformatic tools of type (3) are newer solutions, designed to make organelle assembling easy and robust [38], making them the most used approach.

Due to the circularity of cpDNA and the existence of two SSC orientations, the same cpDNA sequence can be recorded in different forms. Because of a variety of the methods used to assemble sequences, and the fact that public databases do not require a particular form of the cpDNA sequence upon submission [39] (pp. 3–4), it is expected that publicly available cpDNA data will be recorded in many different forms.

Phylogenetics based on cpDNA can be performed (1) on data of one or more genes (e.g., [40,41]), or (2) on data of whole genomes or data of one or more regions (LSC, SSC, IR; e.g., [42,43,44]). In case (1), extracting gene DNA data does not depend on the form in which sequences are stored, and possible incongruences and conflicts between resulting gene trees were analyzed [25,45,46,47]. For case (2), the form of the stored cpDNA whole genome data can influence phylogenetic analyses input data. To our knowledge, there are no studies of the possible influences of the form of stored cpDNA sequences on the results of phylogenetic analyses.

The aim of this research was to investigate the impact of the standardization of cpDNA sequence data on the results of phylogenetic analyses. For this purpose, we collected available cpDNA sequences of five plant families (Apiaceae, Asteraceae, Campanulaceae, Lamiaceae, Rosaceae) and reformatted the sequence data into a strictly defined form. For each family, we created two datasets, the first with the original sequence data and the second with the standardized data. In addition, we created two alignments based on different evolutionary assumptions (with and without partitions). We used two phylogenetic methods (maximum likelihood, Bayesian inference) and compared the trees using four different tree distance methods.

## 2. Results

### 2.1. Input Sequences and Annotations

We acquired all available complete chloroplast genomes for the five families from the NCBI database in December 2020. Statistics of the input data and datasets for each family are presented in Table 1. The families differed considerably in the properties of their datasets. The number of sequences per dataset ranged from 22 (Campanulaceae) to 237 (Asteraceae). Sequence lengths of the datasets ranged from narrow 151.8 ± 2.3 kb (Asteraceae) and 152.5± 2.8 kb (Lamiaceae) to quite wide 145.3 ± 15.6 kb (Rosaceae). Some datasets contained sequences assembled more than 10 years ago (e.g., 2005 Asteraceae and 2006 Apiaceae), while some datasets contained only recent assemblies, the earliest being from 2013 (Lamiaceae) or 2010 (Rosaceae).

### 2.2. Standardization and Alignment

Table 2 shows the number of sequences that disagree with the standard form, including the number of sequences by type of disagreement. Table 2 also shows the properties of the original and standardized datasets, the alignment length, and the number of partitions. The number of sequences that did not match the standard form varied among families, in number and in proportion to the number of sequences analyzed. The number of sequences to be standardized ranged from 7 (Lamiaceae), representing 13% of analyzed sequences, to 172 (Asteraceae), representing 72.6% of analyzed sequences. All datasets contain at least one sequence that has a region in incorrect orientation. The most common region that did not match the standard form was SSC. In all datasets, except for the family Campanulaceae, there was one sequence with a non-matching offset. The maximum offsets found in the datasets varied from a relatively small offset of 1.8 kb (Lamiaceae) to a fairly large one of 68.6 kb (Asteraceae).

In all families except Campanulaceae, the length of the standardized sequence alignment was shorter than the length of the original sequence alignment, which was to be expected. In the standardized alignment of Lamiaceae, the length was only slightly shorter than the original one because the original sequences were already well aligned. The shortening of the alignment varied among families and it appeared that the length of the shortening was related to the alignment length of the original sequences and the number of sequences that needed to be standardized.

Sequence standardization increased the number of partitions (aligned genes) for all datasets. The case of Campanulaceae, where the standardized sequence alignment was longer than that of the original sequences, can be explained by the increased number of partitions, indicating that more genes were successfully aligned after standardization.

**Figure 1:**
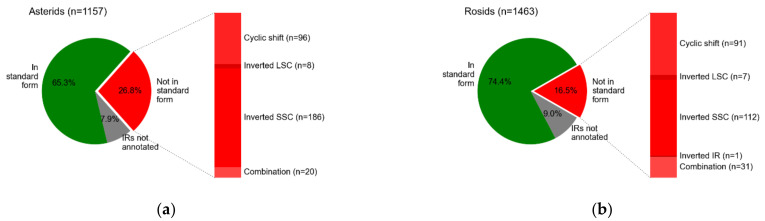
Pie charts showing statistics regarding the forms of cpDNA sequences belonging to asterids (**a**) and rosids (**b**) found in the NCBI database, and annotated with GeSeq. The sequences are classified as follows: sequences without annotated IRs for which an assessment of form is not possible (in grey), sequences that are in standard form (in green), and sequences that deviate from standard form (in red). The third group is additionally classified according to the cause of the deviation from standard form: cyclic shift, inverted LSC, inverted SSC, inverted IRs, and a combination of causes.

Statistics regarding the forms of cpDNA sequences belonging to two major groups of eudicots, asterids, and rosids [48] found in the NCBI database are shown in Figure 1. These two groups account for more than half of the available cpDNA sequences of land plants in the NCBI and annotated with the GeSeq online tool. In both cases, there are a remarkable number of sequences that are not in the standard form (26.8% in asterids, 16.5% in rosids), and the most common deviation from the standard is the different orientation of SSC, followed by cyclic shift.

### 2.3. Tree Comparisons

**Figure 2:**
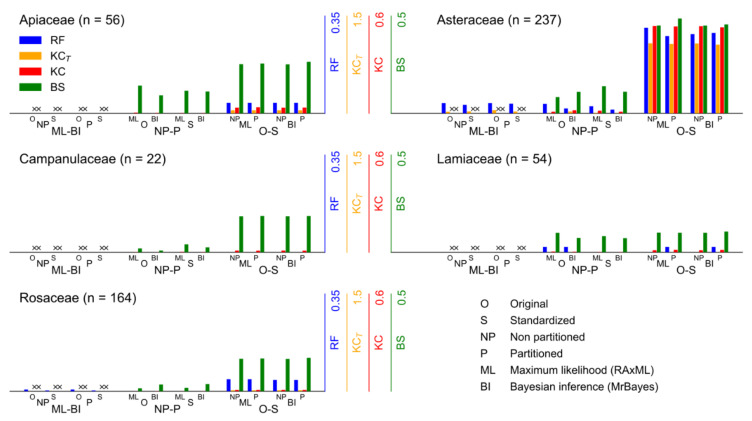
Differences between phylogenetic trees obtained by different phylogenetic methods (maximum likelihood vs. Bayesian inference), by different partitioning schemes (non-partitioned vs. partitioned), and by different sequence data (original vs. standardized), calculated by four tree distance measures (Robinson–Foulds distance, RF; Kendall–Colijn distance with λ = 0, KC_T_, and λ = 0.5, KC; branch score distance, BS) of whole chloroplast genome sequence datasets from five plant families (Apiaceae, Asteraceae, Campanulaceae, Lamiaceae, Rosaceae). Each distance is scaled to the maximum value reached in all comparisons.

The distances between the resulting phylogenetic trees are shown in Figure 2. Tables with the distance values can be found in Supplementary Table S1. The results in the graph are grouped according to the types of comparisons we performed: (1) comparison of different phylogenetic methods, (2) comparison of different partition schemes, and (3) the comparison of different sequence data.

The analyses of the families showed different patterns of results. The results of Apiaceae showed that there were no topological differences in the ML-BI and NP-P trees, and comparisons of NP-P showed some differences in the edge branches. Comparisons of O-S trees showed consistent topological differences in RF (4 of 109 branches) and KC_T_ (73 on 1540 MRCA), with almost the same values for KC and BS distances. In the Asteraceae, there were similar topological differences in the ML-BI and NP-P comparisons. There were greater topological and length differences in the O-S comparisons compared to the other comparisons, as RF distance values were about 10 times greater, KC_T_ more than 20 times, KC more than 30 times, and BS about five times greater. All Campanulaceae trees have the same topology and branch length, O-S distances were four to five times larger than the NP-P distances. Both the ML and BI analyses in Lamiaceae yielded the same tree and the other two comparisons showed minimal differences in topology and branch length. In Rosaceae, ML-BI distances showed small topological differences (RF max. 2 branches out of 323), same topologies for NP-P comparisons, while in O-S comparison all distances were at least five times larger than in the other comparisons.

Comparisons of different phylogenetic methods on the same alignment and partition scheme, in all families except Asteraceae, show that the methods ML and BI lead to exactly the same or slightly different tree topology.

In the second type of comparison, of different partition schemes, the results showed consistent differences in BS distance and for some families, small differences in other distances. When the distance measuring topology and branch lengths (BS) showed differences and the distance measuring only topology (RF) did not, this indicated that the difference between trees was mainly in branch length. For all families, these results showed that the partitioning schemes did not have a large effect on the resulting topology, but they could affect the resulting branch lengths of the trees.

Comparisons of the third type showed an increase in all distances compared to the second type of comparison in all families except Lamiaceae. An increase was notable in Apiaceae, Asteraceae, and Rosaceae, as all distances differed by an order of magnitude.

## 3. Discussion

Most cpDNA genomes have a quadripartite structure with two copies of a large inverted repeat (IR), separated by small (SSC) and large (LSC) single copy regions [8,14].

IR copies facilitate flip-flop recombination, leading to the presence of isoforms that differ in the orientation of single-copy regions [11,12]. Currently, whole chloroplast genomes are published in GenBank with no preference for the orientation of the SSC region [49,50]. This property has been overlooked in several analyses that have evaluated the orientation of the SSC region a phylogenetic context [51,52,53,54,55,56].

### 3.1. Inconsistencies in cpDNA Sequence Data

Due to two properties of the cpDNA genome, namely its presence in two orientations and circularity, coupled with the lack of a strictly defined standard for the linear form of the sequence when posting data to public databases, databases are expected to contain sequence data in different linear forms. Fortunately, most of the sequences retrieved from the NCBI used the form described in the first assembled complete cpDNA genome sequences of *Nicotiana tabacum* L. [6] and *Marchantia polymorpha* L. [5], as shown in this study. Differences in form include the orientation of the SSC and LSC regions and cyclic shifts.

These differences in genome sequence form can be attributed to different tools and approaches in the assembly process. There are two main approaches to cpDNA genome assembly: the use of general whole-genome assemblers and, more recently, the use of targeted assemblers for organelles. General whole-genome assemblers, such as SOAPdenovo [34], ABySS [35], and SPAdes [57], produce one or more contigs depending on the sequencing data input, which must be manually corrected or assembled into a single contig according to the structure of the chloroplast genomes.

Organelle-targeted assemblers such as Fast-Plast [36], GetOrganelle [37], and NOVOPlasty [58] are newer and more efficient methods for generating single-contig cpDNA genome sequences. These assemblers enforce some standardization of their output, but the standardization is not universal [59] (p. 12). Full standardization is implemented in Fast-Plast and is used in this research. The starting position of the sequence and the direction and the order of the regions are maintained in GetOrganelle, while the results consist of two sequences with two versions of the inverse complement of the SSC regions. NOVOPlasty results in one or two sequences in which the regions are arranged in the order IRa, LSC, IRb, and SSC. The resulting sequence of org.ASM [60] has regions in the order SSC, IRa, LSC, and IRb, while chloroExtractor [61] produces a sequence with the LSC region inverted compared to other assemblers. Unlike other targeted assemblers, IOGA [62] can yield multiple contigs, one for each region (LSC, SSC, and IR). This shows that even cpDNA genome sequences assembled with recent specialized tools do not apply a universal standardization of the form.

Nowadays, it is common practice to use publicly available data of whole cpDNA genomes in phylogenetic analyses, especially in research involving assembled genomes (e.g., [63,64,65,66,67,68]). However, it is expected that the use of misaligned, non-homologous data may lead to erroneous results of phylogenetic analyses.

### 3.2. Comparison Procedure

The differences in the alignments of the datasets with original and standardized whole-genome sequences are presented by alignment length and number of genes aligned in the dataset.

To investigate the order of influence on phylogeny, three sets of comparisons were performed using four tree distance methods. The sets of comparisons between phylogenetic outcomes were: (1) between the results of different phylogenetic methods on the same dataset, (2) between the results of analyses of the same datasets with and without gene partitions, (3) between the results of the analyses of the original and standardized sequence data. If comparisons of type (1) show the same or very close topology of the resulting trees, this shows the stability of the results with respect to the phylogenetic methods used, and the resulting tree on the dataset is reliable. The values of the type (2) comparisons give a norm of how much variability to expect in the phylogenetic results when different partition schemes are used. The choice of partition scheme is a step in the phylogenetic analysis that covers our assumptions about the underlying genomic data. Results are expected to differ depending on choice, and if they do, the differences are not critical because the genomic data carry enough information to support the underlying phylogenetic structure. If we relate the comparative values of type (3) to type (2) comparisons, we can see how much the phylogenetic results are affected by working with different forms of cpDNA genomic data. Non-standardized cpDNA whole genome data may contain sequences with SSC regions in both orientations and sequences stored with different starting positions. Consequently, phylogenetic results based on data with this noise are less plausible than results based on data without it.

In this study, we analyzed five family datasets that differ in the number of sequences and sequence lengths. The number of sequences in the dataset varies from 22 (Campanulaceae) to 237 (Asteraceae) sequences, and the percentage of how much longer the maximum length sequence is than the minimum length sequence varies from 3% (Asteraceae) to 24% (Rosaceae).

### 3.3. Impact of Standardization

The alignment lengths of the standardized sequences are shorter than the alignment lengths of the original sequences by 1 (Lamiaceae) to 30% (Asteraceae) in all datasets except Campanulaceae. In the Campanulaceae dataset, the alignment length increased by 2%. The alignment of the standardized data results in more aligned genes than the alignments of the original sequences in all datasets. The number of aligned genes increased by 10 (Lamiaceae) to 263% (Asteraceae).

The longer alignment after data standardization, in the case of the family Campanulaceae, can be explained by the better alignment of the gene regions. The length of the alignment increased by 2%, but the number of aligned genes increased by 64%. The likely reason that standardization does not lead to even more aligned genes or a shortening of the overall alignment is that Campanulaceae species have highly rearranged genomes [69]. Another possible reason can be IR boundary shift [70].

In the case of Lamiaceae, the relatively small increase in the number of aligned genes can be explained by the small number of standardized sequences and by the fact that the alignment of the original sequences aligns most of the annotated genes, namely 88 out of 100–120 unique genes [1]. The standardization of the sequence data of Lamiaceae shows different properties and produces a slightly shorter alignment and a few more aligned genes. This is because the dataset consists of very similar sequences and thus few standardization procedures are required. The reason could be that the dataset consists of sequences that have been recently added, sequenced with newer technologies, and assembled with newer tools that pay attention to cpDNA properties.

Comparisons of the results of different phylogenetic methods on the same dataset, in all families except Asteraceae, show that both phylogenetic methods result in trees with the same or slightly different tree topologies, suggesting that the phylogeny is stable and accurate. In the tree comparisons, the Asteraceae showed different results than the other families for the ML-BI and NP-P comparison types. This can be explained by the much larger dataset, where the space of possible phylogenetic trees is huge, with many resulting trees of similar likelihood.

As expected, comparisons of the results on different partition schemes show some, but not large, differences. The results for the families Apiaceae, Campanulaceae, and Rosaceae differ only in branch length, while the resulting trees for the families Asteraceae and Lamiaceae also differ in topology. The results for the family Lamiaceae showed no differences between O-S and NP-P comparisons. This is not in contradiction with the research results, as the differences are very small in all cases. Small values of all tree distance measures indicate clean genomic data supporting a stable phylogenetic structure where different phylogenetic approaches result in only minor differences at best. The comparison of the resulting phylogenetic trees on the original and standardized sequence data yields value orders of magnitude larger than comparisons on different partition schemes in all families except Lamiaceae.

We can conclude that in general (a) standardization leads to a shorter alignment, a higher number of aligned genes, and most likely a better tree, and that (b) standardization has a significantly greater impact on phylogenetic analyses than the choice of partition scheme.

We therefore recommend standardizing cpDNA genome sequence data before analysis and provide a Python script for this purpose (https://github.com/CroP-BioDiv/cpDNA_standardization (accessed on 30 May 2021)).

## 4. Materials and Methods

### 4.1. Data Acquisition

Complete chloroplast genome sequences were downloaded from the NCBI (http://www.ncbi.nlm.nih.gov (accessed on 20 December 2020)). We acquired all genomes available at that time for asterids and rosids clades. In total 2620 sequences were downloaded. Of these data, datasets from five plant families (Apiaceae, Asteraceae, Campanulaceae, Lamiaceae, and Rosaceae) were constructed. The criteria for selecting families were to work with relatively close families whose dataset properties differed. The selected families differed in dataset size and range of sequence lengths. Some datasets contained only recently assembled sequences, while some included sequences uploaded more than 10 years ago. For some species, more than one genome could be found in the NCBI. In all cases, multiple genomes of the same species belonged to different subspecies and we decided to use the most recent genome for a species. To follow standard procedures in phylogenetic analyses, an outgroup sequence was included for each family.

### 4.2. Standard Form of Whole Chloroplast Genome Sequence

The chloroplast genome sequence can be stored in many forms. We have chosen the notation of the cpDNA sequence, here referred to as the standard form, following the most commonly used notation derived from the first two available land plant cpDNA complete genome sequences, *Nicotiana tabacum* L. [6] and *Marchantia polymorpha* L. [5].

The origin of the sequence was the boundary between the LSC and IRa regions. The sequence direction was toward the LSC. The order of regions was: LSC, IRb, SSC, and IRa. The orientation of the sequence regions was determined by checking the relative orientation of the characteristic genes on these regions, as described in the comment on Fast-Plast [71]. The orientation of the LSC was determined by checking the *rpl* and *rps* genes, SSC by checking all genes, and IRb by checking the *rrn* genes. For each region, more genes should be on the negative strand than on the positive strand.

For sequences whose annotation did not contain inverted repeats, it was impossible to infer the relationship of the sequence to the standard form. Two procedures were possible to convert any sequence to standard form: (1) replacing one or more sequence regions (LSC, SSC, IRs) with the inverse complement; (2) cyclically shifting the entire sequence by cutting part of a sequence at the end and inserting it at the beginning, or vice versa. We set the minimum value for the cyclic shift offset to 10 bp. The offset value was set to 10 bp because assemblies based on short-read sequence data sometimes incorrectly assign the ends of the LSC and SSC regions to the IR region and vice versa, for ~10 bp [72].

### 4.3. Sequence Annotation and Standardization

Chloroplast genome sequences downloaded from the NCBI already contain annotations. These sequences have been annotated using different tools and different versions of tools, while some were manually edited. To work with consistent annotations, we annotated all genome sequences using GeSeq [73], as the tool is recent and continuously maintained, and there is evidence it is more consistent in the detection and annotation of genes than some other tools [74,75].

Sequences without annotated inverted repeats were omitted from further analyses. For each family, we created two datasets, the first consisting of data from original genome sequences acquired from the NCBI and the second consisting of the same data in standard form.

We designed a pipeline that included the following steps: acquisition of input data, annotating sequences, analysis of sequence data, reformatting into standard form, annotating sequences changed by standardization, alignment and phylogenetic analyses, comparisons of phylogenetic results, and representation of comparisons. The pipeline was implemented in Python using the Biopython package [76]. The code is maintained in a public repository (https://github.com/CroP-BioDiv/zcitools (accessed on 30 May 2021)).

### 4.4. Tree Constructions and Comparisons

For each plant family, we created two alignments: one alignment from original sequences (original; O) and one from the same sequences in standardized form (standardized; S). For each alignment, we created two datasets: one without partitioning (non-partitioned; NP) and one with partitioning created from the corresponding annotation data (partitioned; P). On each dataset, we performed two types of phylogenetic analyses, maximum likelihood (ML) and Bayesian inference (BI). For both phylogenetic analyses, we used a generalized time-reversible (GTR) substitution model with gamma-distributed rates over nucleotide sites.

**Figure 3:**
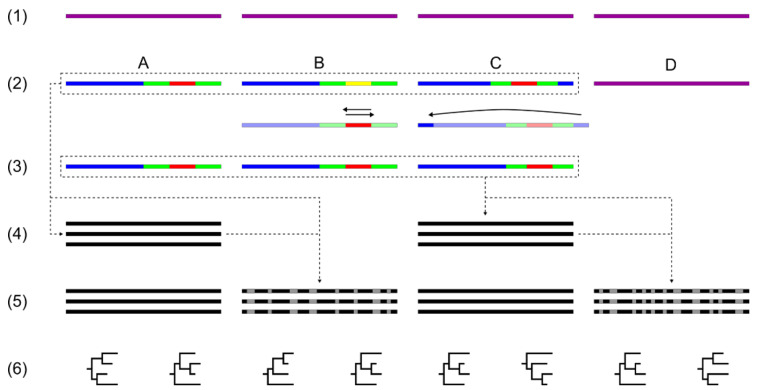
Flowchart showing the analysis steps for the input data of a family. Workflow: (**1**) Input: input sequences collected from the NCBI; (**2**) Annotation: sequence annotation using GeSeq; (**3**) Standardization: sequence standardization and annotation of changed sequences; (**4**) Alignment: alignment of original and standardized sequences; (**5**) Partitioning: creation of partitions used in phylogenetic analyses; (**6**) Phylogeny: phylogenetic analyses (BI and ML) on non-partitioned and partitioned alignments. Sequences: (**A**) sequence in standard form; (**B**) sequence with a region (SSC) reversed to standard form; (**C**) sequence with a shift; (**D**) sequence without annotated IRs. Chloroplast regions are indicated by different colors: whole sequence (purple); LSC (blue); IRa and IRb (green); SSC (red, yellow); alignment (black); gene segments (grey).

Alignment was performed with MAFFT v. 7.453 [77], using the iterative refinement method strategy (FFT-NS-i), with maximum refinement cycles set to 10 (--maxiterate 10). Maximum likelihood (ML) analyses were performed using RAxML v. 8.2.12 [78], assuming the GTR + Γ model of substitution (-m GTRGAMA). Bootstrap support was estimated from 1000 replicates (-f a -N 1000). Bayesian inference was performed using MrBayes v. 3.2.7 a [79,80]. The likelihood model parameters of MrBayes used were “nst=6 rates=gamma”, the MCMC parameters used were “ngen=300000 samplefreq=300 nchains=4”, and the summary parameters included “relburnin=yes burninfrac=0.25”. The choice of the number of generations in MCMC was determined by a trade-off between the computational time and the accuracy of the results. The alignments and phylogenetic calculations were carried out on the local server and computational cluster “Isabella” within the University of Zagreb Computing Center (SRCE). A flowchart showing the analysis steps is depicted in Figure 3.

The results of the phylogenetic analyses (trees) were compared with three distances: (a) Robinson–Foulds distance (RF) [81], (b) Kendall–Colijn distance (KC) [82], and (c) Branch score distance (BS) [83]. The Python library ETE toolkit [84] was used to calculate RF distance and to implement the methods for calculating KC and BS distances. Tree distance measures how much two trees differ in topology only, or in topology including branch lengths. For distances that measure differences in both topology and branch lengths, it is difficult to tell from the distance value how much differences in topology and differences in branch lengths contribute to the final result. We used several distances calculated in different ways to analyse differences in both properties. RF distance measures only topology differences, while KC and BS distances measure differences in both topology and branch lengths. KC distance depends on a predefined weighting parameter λ. We used two variants of KC distance: KC_T_ with λ = 0, which measures only topological differences, and KC with λ = 0.5, which measures differences in both traits. The values of the different distances are not comparable.

The comparison of measurements of the same distance does not depend on the scale. For all distances, the maximum possible measurement depends on the properties that the distance takes into account, and these properties depend on the number of sequences analyzed. As the number of sequences in the family data sets varied widely, from 22 to 237 sequences, we scaled the measured distances for better readability. The distance values were scaled by a factor depending on the number of properties the distance measures: RF was scaled by the number of branches, KC_T_ was scaled by the number of distinct sequence pairs, KC was scaled by the number of distinct sequence pairs plus the number of sequences, and BS was scaled by the sum of branch lengths in both trees. The plot of tree distances (Figure 2) was created using the Python library Matplotlib [85].

We have made three types of comparisons. Firstly (1), we compared different phylogenetic methods on the same alignment and partition scheme. We analyzed four data sets (O/NP, O/P, S/NP, S/P) using both maximum likelihood and Bayesian inference methods. Since the branch lengths between resulting trees of different phylogenetic methods were not comparable, it was possible to compare only the topology of the trees (RF, KC_T_). It was expected that the topologies of the resulting trees of the same dataset would be the same or very similar. Secondly (2), we compared different partition schemes on the same alignment. For four combinations (O/BI, O/ML, S/BI, S/ML), we compared resulting trees generated on non-partitioned and partitioned alignments. Since the comparison was made with trees generated using the same phylogenetic method, it was possible to compare both topology and branch lengths (RF, KC_T_, KC, and BS). It was expected that the resulting trees would be similar. Thirdly (3), we compared different sequence data. For four combinations (NP/BI, NP/ML, P/BI, P/ML) we compared resulting trees generated from original and standardized sequences. The comparison was performed on trees generated by the same phylogenetic method, using all distance methods (RF, KC_T_, KC, and BS). We wanted to check how the distances of this comparison type would relate to the distances of the first two comparison types.

## Figures and Tables

**Table 1:** Input data and dataset statistics: numbers of acquired cpDNA whole genome sequences, outgroup used in phylogenetic analysis for five families (Apiaceae, Asteraceae, Campanulaceae, Lamiaceae, Rosaceae), overall numbers of sequences in datasets included in the analyses, and statistics for each family dataset.

Parameter	Family				
	Apiaceae	Asteraceae	Campanulaceae	Lamiaceae	Rosaceae
No. of NCBI genomes	58	259	30	62	181
No. of different species	58	252	30	62	178
Outgroup species	*Aralia continentalis* Kitag.	*Adenophora triphylla* (Thunb.) A.DC.	*Artemisia ordosica* Krasch.	*Pedicularis longiflora* (Klotsch.) P.C.Tsoong.	*Ulmus elongata* L. K. Fu & C. S. Ding
Outgroup accession number	NC_041648	NC_040857	NC_046571	NC_046852	NC_046061
No. of sequences included in the analyses (with outgroup)	56	237	22	54	164
Min. genome length (bp)	141,948	149,510	151,061	149,736	129,788
Max. genome length (bp)	164,653	154,223	176,331	155,293	160,937
Earliest NCBI create date	6 September 2006	8 November 2005	25 March 2008	2 January 2013	2 December 2010
Latest NCBI create date	10 December 2020	10 December 2020	28 June 2020	10 December 2020	10 December 2020

**Table 2:** Standardization and dataset alignment results. Number of cpDNA whole genome sequences used in phylogenetic analyses for five families (Apiaceae, Asteraceae, Campanulaceae, Lamiaceae, Rosaceae), with numbers of sequences not in standard form detailed by numbers of sequences in which one or more sequence regions were replaced with the inverse complement and number of cyclically shifted sequences. Alignment length and number of gene partitions is shown for both family datasets of sequences (original and standardized).

Parameter	Apiaceae	Asteraceae	Campanulaceae	Lamiaceae	Rosaceae
No. of sequences included in the analyses	56	237	22	54	164
No. of standardized sequences ^1^	9	172	8	7	14
No. of sequences in which one or more sequence regions were replaced with the inverse complement	1	170	8	1	3
• Sequence regions replaced with the inverse complement	SSC	SSC	7 LSC, 1SSC	SSC	SSC
No. of sequences which were cyclically shifted	8	11	0	6	11
• Offset range (bp)	18–40,289	14–68,671	-	101–1803	30–29,019
Alignment length of the original sequences	234,804	302,303	308,697	184,539	264,556
• No. of gene partitions	48	22	14	88	40
Alignment length of the standardized sequences	204,800	210,475	315,141	182,261	208,977
• No. of gene partitions	88	80	23	97	62

^1^ The number of standardized sequences is equal to or less than the sum of reoriented and cyclically shifted sequences, since some sequences were both reoriented and shifted.

## Data Availability

The data that support the findings of this study are openly available in GenBank at the NCBI (https://www.ncbi.nlm.nih.gov (accessed on 20 December 2020)). Complete research data and results are archived as publicly available dataset (https://doi.org/10.5281/zenodo.4807017 (accessed on 30 May 2021)).

## Supplementary Materials

The following are available online at https://www.mdpi.com/article/10.3390/plants10071360/s1, Table S1: List of GenBank accession numbers, and data collected, for the cpDNA sequences used in the analyses; Table S2: Tree distances presented in Figure 2.
